# Sexual Violence at University: Are Varsity Athletes More at Risk?

**DOI:** 10.3389/fpsyg.2022.861676

**Published:** 2022-04-25

**Authors:** Sylvie Parent, Isabelle Daigneault, Stephanie Radziszewski, Manon Bergeron

**Affiliations:** ^1^Research Chair in Security and Integrity in Sport, Université Laval, Québec City, QC, Canada; ^2^Department of Physical Education, Faculty of Education, Université Laval, Québec City, QC, Canada; ^3^International Research Network on Violence and Integrity in Sport (IRNOVIS), Antwerp, Belgium; ^4^Interdisciplinary Research Center on Intimate Relationship Problems and Sexual Abuse (CRIPCAS), Montréal, QC, Canada; ^5^Équipe Violence Sexuelle et Santé (ÉVISSA), Université du Québec à Montréal, Montréal, QC, Canada; ^6^Department of Psychology, Université de Montréal, Montréal, QC, Canada; ^7^Research Chair on Sexist and Sexual Violences in Post-secondary Education Institutions, Université du Québec à Montréal, Montréal, QC, Canada; ^8^Department of Psychology, Université du Québec à Montréal, Montréal, QC, Canada; ^9^Department of Sexology, Université du Québec à Montréal, Montréal, QC, Canada

**Keywords:** sexual violence, varsity athletes, university, sport protection hypothesis, routine activity theory

## Abstract

Some studies report that the sport context increases the risk of exposure to sexual violence for athletes. In contrast, others indicate a protective effect of sport participation against sexual violence, particularly among varsity athletes. Studies of sexual violence towards varsity athletes are limited by their failure to include control groups and various known risk factors such as age, graduate level, gender and sexual identity, disability status, international and Indigenous student status, and childhood sexual abuse. The purpose of the present study is to fill in these gaps to determine whether varsity athletes are at greater risk than non-athletes of sexual violence towards them or whether, on the contrary, involvement in a varsity sport is coherent with the Sport Protection Hypothesis. Data for this article come from the ESSIMU study (*Enquête sur la Sexualité, la Sécurité et les Interactions en Milieu Universitaire*), a broad survey of students, professors, and other employees at six francophone universities regarding sexual violence on university campuses. A total of 6,485 students with complete data on sexual violence, athlete status, and gender were included in the study. From this total, 267 participants identified themselves as varsity athletes. Data were analyzed using a series of logistic regressions on each form of violence using athlete status as a predictor and characteristics associated with sexual violence victimization or distinguishing between varsity athletes and non-athletes as confounding variables. When considering all confounding variables in the regression analyses on four yearly incidence rates of sexual violence, the results revealed that being a varsity athlete did not significantly increase the risk of exposure to sexual violence at university. All considered other variables were more significant predictors of the past year’s risk of sexual violence victimization than athlete status was.

## Introduction

Sport is recognized as a positive development tool for young people (Holt and Neely, 2011). In addition, various studies have shown the positive or protective effects of sport participation, whether for physical or mental health (Eime et al., 2013; Jewett et al., 2014) or rehabilitation (Rioux et al., 2020). Some studies point out that sport can also promote resilience in young people exposed to various childhood forms of adversity (Romans et al., 1995; Asgeirsdottir et al., 2010). However, the links between sport participation and sexual violence are sometimes contradictory. Some seminal work stated that the sport context is conducive to sexual violence towards athletes (e.g., Brackenridge, 2001; Hartill, 2016). In contrast, other studies indicate a protective effect of sport participation against sexual violence throughout life (Parent et al., 2016), particularly among varsity athletes (Fasting et al., 2008). Fasting, Brackenridge, and Sundgot-Borgen first proposed an explanation of this “protection” effect by suggesting the Sport Protection Hypothesis in 2003. This hypothesis is based on the belief that athletes “develop strength, self-confidence, and a sense of physical adeptness through their sports experiences” (Fasting et al., 2003, p. 428) and that this could provide a form of protection or defense against sexual violence. However, studies on sexual violence and sport in university settings have mainly focused on the risk of athletes committing sexual violence rather than being exposed to it. Consequently, the current body of knowledge does not allow a clear understanding of sport participation’s role in sexual violence in the university context. Studies of sexual violence towards varsity athletes are also limited by their failure to include control groups and various known risk factors for sexual violence in a university context such as age, graduate level, gender and sexual identity, disability status, international or Indigenous student status, and childhood sexual abuse. The following sections describe the state of knowledge on this issue.

### Sexual Violence and Varsity Athletes

The literature on sexual violence in a university context has more often reported the risk posed by athletes as perpetrators (McCray, 2019; Bonar et al., 2020) than the risk of sexual violence towards varsity athletes. In a recent review of the literature on risk factors associated with sexual violence in the university setting, Bonar et al. (2020) concluded that being a member of a men’s university athletic team was a risk factor for sexual assault perpetration. Murnen and Kohlman (2007) explained that this could be due to hypermasculinity values and level of aggressiveness in male sport, those problematic values and behaviors being associated with sexual violence. Indeed, it appears that male athletes adhered to more rape myths and held more rape-supportive attitudes than non-athletes (Boeringer, 1999; Sawyer et al., 2002; Young et al., 2017). In contrast, studies that have examined the adoption of sexually violent behaviors are not as straightforward as those linking male athlete status and rape-supportive attitudes (Murnen and Kohlman, 2007; Tharp et al., 2013). Boeringer (1996) found that athletes did not engage in sexual violence behaviors to a greater extent than non-athletes in a university setting. Furthermore, another study identified that membership in a sports team contributed to explaining only 1% of the variance in sexual assault in a university setting (Koss and Gaines, 1993). Conversely, Young et al. (2017) showed that college athletes reported higher rates of sexual coercion than non-athletes.

While these findings are contradictory and deserve further research, we can question whether the university sports environment represents a risk factor for victimization for athletes. In this regard, Bonar et al. (2020) concluded that male peer affiliation such as with an athletic team represents a risk factor for the perpetration of sexual violence in the university environment. Given that some male athletes may more likely be perpetrators, their peer athletes may be at increased risk because they more often interact with them. The Routine Activity Theory (Cohen and Felson, 1979) have been used in the past in sexual violence victimization studies (e.g., Cass, 2007; Snyder, 2015; Davis et al., 2021) and could explain this potentially elevated risk for athletes. This theory explains the occurrence of crime by three contributing factors. In the case of sexual violence, those would be a motivated perpetrator (i.e., male peer athletes), a suitable target (other athletes), and an absence of a capable guardian. For the last factor, we know that the sport context has often been singled out for its institutional tolerance of sexual violence and other forms of maltreatment against athletes (Jacobs et al., 2017; Parent and Fortier, 2018; Roberts et al., 2020). Based on those factors and the literature presented above, there could be more perpetrators in the sport environment, or a lack of capable guardians given the greater adherence to rape myths in the masculine sport environment.

#### Varsity Athletes as Victims

As already mentioned, several studies have focused on sexual violence perpetrated by varsity athletes towards other students on campus (see McCray, 2015 for a review), but, to our knowledge, none has explicitly focused on the sexual violence against them in a university context. Research with samples of university student-athletes has mainly focused on harassment or sexual assault of student-athletes throughout their life or their sporting career (e.g., Chroni and Fasting, 2009; Ahmed et al., 2018). The magnitude of exposure to sexual violence in the student-athlete population varies between 21 and 64.4% (Van Niekerk and Rzygula, 2010; Fasting et al., 2011, 2014; Rintaugu et al., 2014; Ahmed et al., 2018). The perpetrators of this form of violence are overwhelmingly peer athletes (Elendu and Umeakuka, 2011; Rintaugu et al., 2014). Fasting et al. (2008) reported that 6.9% of their sample of American university student-athletes mentioned having been forced to have sexual relations in their lifetime. Even though we do not have explicit knowledge of what varsity athletes are exposed to in a university context in terms of sexual violence, research shows that sexual violence on university campuses affects a significant proportion of students (Banyard et al., 2007; Krebs et al., 2007; Fedina et al., 2018; Bergeron et al., 2019; Moylan and Javorka, 2020). Varsity athletes are certainly not spared from the problem, but our knowledge in this regard is minimal.

However, we know that some factors increase the risk of sexual violence against athletes more broadly. For example, we know that athletes who identify as a sexual minority report more sexual violence (Vertommen et al., 2016; Parent and Vaillancourt-Morel, 2021). Studies related to gender are inconclusive and vary depending on the sample and methodology used. Some found differences, showing that female athletes reported more sexual violence (Vertommen et al., 2016; Ohlert et al., 2021), and others did not, showing that female and male athletes reported the same level of sexual victimization (Fasting et al., 2008; Parent et al., 2016; Parent and Vaillancourt-Morel, 2021). Age, level of performance, disability status, and type of sport are characteristics that are also still inconclusively associated with an increased risk of sexual violence against athletes across studies (e.g., see Vertommen et al., 2016; Ohlert et al., 2021; Parent and Vaillancourt-Morel, 2021). One risk factor has frequently been reported in the literature on sexual violence against athletes: the power imbalance (formal and informal) between individuals and the potential it creates for situations of abuse of power, such as in coach-athlete and rookie-veteran relationships (Parent and Fortier, 2018; Roberts et al., 2020). This means that, in the university sport context, it could also be possible that varsity athletes are at risk of being exposed to sexual violence within these relationships. For example, abusive team cohesion events intended to initiate rookies (namely “hazing”) are situations where sexual violence and other forms of violence are reported (Crow and Macintosh, 2009). Also, situations of sexual abuse from coaches are reported in many studies (Toftegaard Nielsen, 2001; Sanderson and Weathers, 2020; Wilinsky and McCabe, 2021). Despite knowing that varsity athletes could be at risk of sexual violence towards them in a university context based on our knowledge of existing risk factors in sports, it is unclear if this context is a risk or protective factor. The risk factor hypothesis is supported by the Routine Activity Theory (Cohen and Felson, 1979), in contrast to the Sport Protection Hypothesis (Fasting et al., 2008; Parent et al., 2016).

### The Sport Protection Hypothesis

Fasting, Brackenridge, and Sundgot-Borgen first proposed the Sport Protection Hypothesis in 2003. This hypothesis is based on the belief that athletes “develop strength, self-confidence, and a sense of physical adeptness through their sports experiences” (Fasting et al., 2003, p. 428) and that this could provide a form of protection or defense against sexual violence. In 2008, Fasting et al. (2008) tested the Sport Protection Hypothesis, stating that university student-athletes somehow benefited from some form of protection against sexual aggression (penetrative sexual intercourse against their will) compared to other students. The results supported this hypothesis by documenting that university student-athletes (women and men) reported significantly less sexual violence during their late high school and early college years than non-athletes. The authors explained this phenomenon by two main factors or processes: (1) victims of sexual violence may be less likely to join a university sports program, or (2) a sports program or the practice of a sport inherently protect athletes from being exposed to this form of violence (Fasting et al., 2008). These results were corroborated by the work of Parent et al. (2016), who showed that the odds of lifetime prevalence of sexual abuse were 1.32 times higher for adolescents not involved in organized sports than those who were. The results of Parent et al. (2016) and those of Fasting et al. (2008) suggest that participating in sports is a protective factor against the lifetime prevalence of exposure to sexual violence.

Although innovative, these results remain fragmentary and merit further research to counteract some limitations. For example, because they tested the Sport Protection Hypothesis using lifetime prevalence rates of sexual abuse and current athlete status, there is a risk of confusing cause and effect or the timing of events. Since childhood sexual abuse can occur long before becoming an athlete, sport participation may not be protective. Instead, it may be that those with past abuse are less likely to become athletes. To test the Sport Protection Hypothesis, we should thus strive to document sexual violence exposure while being an athlete. Clarifying this antecedent would improve current designs. Another important limitation of these studies is the lack of control for known risk factors for sexual violence victimization in the university context to account for potential confounding variables. These should include belonging to an ethnic, sexual or gender minority (Martin-Storey et al., 2018; Eisenberg et al., 2021; Klein and Martin, 2021), having a disability (Bergeron et al., 2016), being an international or Indigenous student (Dion et al., 2021; Fethi et al., under review)^1^ or having been sexually abused as a child (CSA) (Bergeron et al., 2016).

### Aim of the Study

Studies on sexual violence and sport in university settings have mainly focused on the risk of athletes committing sexual violence rather than being exposed to it. Therefore, the current body of knowledge does not allow a clear understanding of the role that sport participation plays in sexual violence in the university context. Studies of sexual violence against varsity athletes are also limited by their failure to include control groups and various known risk factors and their sole focus on lifetime exposure to sexual violence. In addition, they sometimes document childhood abuse and sexual violence in the university context indiscriminately. The purpose of this study is to fill the gaps from previous studies and determine whether varsity sport participation is a risk or a protective factor for sexual violence victimization in university. We will test these hypotheses while controlling for other known risk factors for sexual violence victimization to ensure that if a risk exists it is not better accounted for by confounding factors. A secondary objective is to determine whether the context of violence differs between varsity athletes and non-athletes.

## Materials and Methods

### Procedures and Participants

The data for this article come from the ESSIMU study (*Enquête sur la Sexualité, la Sécurité et les Interactions en Milieu Universitaire*) conducted in Québec, a broad survey of students, teaching staff and other employees at six francophone universities regarding sexual violence on university campuses (Bergeron et al., 2016). Varied strategies were used to recruit the sample. The principal one was a massive email invitation to answer the online questionnaire sent to the entire university community, using the institutional email lists. The sole criterion for participating in the survey was to be employed or studying at one of the six universities during data collection (January to May 2016). The survey obtained the approval of human research ethics committees at the universities in question. The overall sample consisted of 9,284 participants. A total of 6,485 students with complete data on sexual violence, athlete status, and gender were included in the present study. From this total number of students, 267 identified themselves as varsity athletes. Descriptive statistics about the final sample of participants are presented in Table 1 in the Results section.

**TABLE 1:** Sociodemographic comparisons between varsity athletes and non-athletes.

	Varsity Athletes	Non-athletes			
Total sample	*n* (%)	*n* (%)	χ^2^	*P*	Cramér’s V^b^
Age (*n* = 6,369)			34.347	0.000	0.392
18–25	215 (82%)	3,919 (64%)			
26–35	41 (16%)	1,622 (27%)			
36+	7 (3%)	565 (9%)			
Undergraduate (*n* = 6,485)	211 (79%)	4,014 (65%)	23.614	0.000	0.060
Gender identity^a^ (*n* = 6,485)			2.601	0.272	0.020
Female	187 (70%)	4,602 (74%)			
Male	76 (29%)	1,504 (24%)			
Non-binary/other	–	–			
Sexual identity (*n* = 6,455)			2.951	0.229	0.021
Heterosexual	217 (81%)	5,222 (84%)			
Sexual minorities	43 (16%)	871 (14%)			
Uncertain/questioning	7 (3%)	95 (2%)			
Disability status (*n* = 6,450)			0.181	0.913	0.005
Yes	28 (11%)	650 (11%)			
No	229 (86%)	5,318 (86%)			
Uncertain	8 (3%)	217 (4%)			
Visible minority (*n* = 6,410)	16 (6%)	467 (8%)	0.890	0.346	0.012
Indigenous (*n* = 6,458)	39 (15%)	171 (3%)	114.801	0.000	0.133
International status (*n* = 6,469)	184 (69%)	575 (9%)	879.258	0.000	0.369
Childhood sexual abuse (*n* = 6,485)					
Yes	63 (24%)	1,441 (23%)	0.786	0.675	0.004
No	186 (70%)	4,433 (71%)			
Rather not answer	18 (7%)	344 (6%)			
**Sexual Violence Victimization at university (*n* = 6,485)**					
Sexual Harassment	102 (38%)	2,027 (33%)	3.645	0.056	
Unwanted Sexual Behavior	52 (20%)	1,130 (18%)	0.291	0.589	
Sexual Coercion	12 (5%)	185 (3%)	2.006	0.157	
Sexual Violence (total)	111 (42%)	2,234 (26%)	3.534	0.060	
**Sexual Violence Victimization at university – past 12 months (*n* = 6,485)**					
Sexual Harassment	86 (32%)	1,523 (25%)	8.171	0.004	
Unwanted Sexual Behavior	37 (14%)	762 (12%)	0.609	0.435	
Sexual Coercion	10 (4%)	91 (2%)	8.694	0.003	
Sexual Violence (total)	93 (35%)	1,692 (27%)	7.452	0.006	

*^a^Cell sizes for this category were too small for comparison.*

*^b^For effect sizes regarding sexual violence, please refer to the odds ratios related to the regression analyses in Table 2.*

**TABLE 2:** Logistic regression analyses of university-based sexual violence in the last 12 months (*n* = 6,113).

	Three dimensions combined					Sexual harassment				
	*B* (SE)	Wald χ^2^	*P*	*OR*	95% CI	*B* (SE)	Wald χ^2^	*P*	*OR*	95% CI
Constant	−0.76 (0.15)	316.46	<0.001			−2.95 (0.17)	319.95	<0.001	0.05	
Age (36+ – indicator)		78.24	<0.001				70.75	<0.001		
18–25	1.12 (0.14)	63.15	<0.001	3.06	[2.32, 4.04]	1.19 (0.15)	61.29	<0.001	3.28	[2.44, 4.42]
26–35	0.70 (0.15)	22.66	<0.001	2.02	[1.51, 2.70]	0.83 (0.16)	27.53	<0.001	2.30	[1.68, 3.13]
Graduate (undergraduate – indicator)	−0.13 (0.07)	3.51	0.061	0.87	[0.76, 1.01]	−0.15 (0.07)	4.04	0.044	0.86	[0.75, 1.00]
Gender identity (male – indicator)	0.51 (0.08)	42.16	<0.001	1.66	[1.42, 1.93]	0.51 (0.08)	39.76	<0.001	1.67	[1.42, 1.96]
Sexual identity (heterosexual – indicator)		58.63	<0.001				56.65	<0.001		
Sexual minorities	0.61 (0.08)	52.83	<0.001	1.83	[1.56, 2.16]	0.61 (0.09)	50.81	<0.001	1.83	[1.55, 2.16]
Uncertain/questioning	0.64 (0.22)	8.46	0.004	1.90	[1.23, 2.92]	0.65 (0.22)	8.57	0.003	1.92	[1.24, 2.97]
Disability (no – indicator)		11.30	0.004				10.59	0.005		
Yes	0.30 (0.10)	10.09	0.001	1.35	[1.12, 1.63]	0.30 (0.10)	9.61	0.002	1.35	[1.12, 1.63]
Uncertain	0.22 (0.16)	1.86	0.173	1.24	[0.91, 1.70]	0.20 (0.16)	1.58	0.208	1.23	[0.89, 1.68]
Visible minority	0.01 (0.12)	0.01	0.945	1.01	[0.80, 1.27]	−0.04 (0.12)	0.11	0.738	0.96	[0.76, 1.22]
Indigenous	0.26 (0.16)	2.57	0.109	1.30	[0.94, 1.79]	0.23 (0.17)	1.95	0.163	1.26	[0.91, 1.75]
International status	0.37 (0.10)	13.48	<0.001	1.45	[1.19, 1.76]	0.26 (0.10)	6.30	0.012	1.30	[1.06, 1.59]
Childhood sexual abuse (no – indicator)		223.08	<0.001				188.68	<0.001		
Yes	1.75 (0.13)	187.51	<0.001	5.78	[4.50, 7.43]	1.52 (0.13)	148.42	<0.001	4.58	[3.58, 5.84]
Uncertain / rather not answer	0.57 (0.07)	63.36	<0.001	1.76	[1.53, 2.03]	0.60 (0.07)	68.28	<0.001	1.83	[1.56, 2.11]
Athlete status	0.04 (0.16)	0.08	0.784	1.04	[0.77, 1.42]	0.16 (0.16)	0.97	0.324	1.17	[0.86, 1.59]

Model	$\chi_{\left(14\right)}^{2}$ = 316.46, *p*<0.001					$\chi_{\left(14\right)}^{2}$ = 112.14, *p*<0.001				
	Pseudo R2 (Nagelkerke) = 0.124					Pseudo R2 (Nagelkerke) = 0.113				
	Log likelihood = 6,578.32					Log likelihood = 6294.42				

	**Unwanted sexual behavior**					**Sexual coercion**				
		
	***B* (SE)**	**Wald χ^2^**	***p***	***OR***	**95% CI**	***B* (SE)**	**Wald χ^2^**	***p***	***OR***	**95% CI**

Constant	−3.68 (0.22)	291.28	<0.001	0.03		−5.34 (0.50)	112.14	<0.001	0.01	
Age (36+ – indicator)		51.45	<0.001				7.33	0.026		
18–25	0.93 (0.19)	53.52	<0.001	2.54	[1.74, 3.70]	0.57 (0.44)	1.66	0.197	1.77	[0.74, 4.23]
26–35	0.25 (0.21)	1.40	0.236	1.28	[0.85, 1.92]	−0.30 (0.52)	0.34	0.560	0.74	[0.27, 2.03]
Graduate (undergraduate – indicator)	−0.13 (0.10)	1.85	0.173	0.88	[0.72, 1.06]	−0.35 (0.28)	1.63	0.202	0.70	[0.41, 1.21]
Gender Identity (male – indicator)	0.74 (0.12)	40.08	<0.001	2.10	[1.67, 2.64]	0.34 (0.30)	1.30	0.254	1.41	[0.78, 2.54]
Sexual identity (heterosexual – indicator)		17.90	<0.001				2.17	0.339		
Sexual minorities	0.45 (0.11)	17.84	<0.001	1.57	[1.28, 1.93]	0.39 (0.26)	2.17	0.141	1.47	[0.88, 2.46]
Uncertain/questioning	0.17 (0.29)	0.32	0.569	1.18	[0.67, 2.10]	–^a^	–	–	–	–
Disability (no – indicator)		7.79	0.020				4.53	0.104		
Yes	0.30 (0.12)	6.30	0.012	1.35	[1.07, 1.71]	0.58 (0.28)	4.39	0.036	1.78	[1.04, 3.06]
Uncertain	0.29 (0.20)	2.16	0.142	1.33	[0.91, 1.95]	−0.09 (0.60)	0.02	0.879	0.91	[0.28, 2.30]
Visible minority	0.04 (0.15)	0.07	0.796	1.04	[0.77, 1.40]	0.37 (0.35)	1.14	0.285	1.45	[0.73, 2.88]
Indigenous	0.09 (0.22)	0.16	0.691	1.09	[0.71, 1.67]	−0.74 (0.73)	1.02	0.313	0.48	[0.11, 2.01]
International status	0.58 (0.13)	21.42	<0.001	1.79	[1.40, 2.29]	0.18 (0.35)	0.26	0.607	1.20	[0.61, 2.37]
Childhood sexual abuse (no – indicator)		80.69	<0.001				20.20	<0.001		
Yes	1.13 (0.14)	62.86	<0.001	3.11	[2.35, 4.11]	0.97 (0.38)	6.70	0.010	2.64	[1.27, 5.51]
Uncertain	0.54 (0.09)	33.39	<0.001	1.71	[1.43, 2.05]	1.00 (0.23)	18.18	<0.001	2.71	[1.71, 4.28]
Athlete status	−0.39 (0.21)	3.32	0.069	0.68	[0.45, 1.03]	0.74 (0.43)	2.95	0.086	2.10	[0.90, 4.89]

Model	$\chi_{\left(14\right)}^{2}$ = 319.95, *p*<0.001					$\chi_{\left(14\right)}^{2}$ = 112.14, *p*<0.001				
	Pseudo R2 (Nagelkerke) = 0.087					Log likelihood = 893.54				
	Log likelihood = 4,209.50					Log likelihood = 893.54				

*^a^This category was invariant across groups and estimates are unstable/invalid.*

### Measures

The complete questionnaire was composed of 13 sections and took an average of 15–20 minutes to answer. The two sections of interest for the present study were the sociodemographic measures described below and the sexual violence in the university context survey.

#### Varsity Athlete Status

Athlete status was assessed using the following question: “Are you part of an official university sports team as an athlete?” Participants answered on a yes/no basis.

#### Confounding Risk Factors

Nine self-reported participant characteristics known as risk factors were assessed and used as confounding variables in analyses. **Age** was determined by one question with seven possible answers (less than 17 years old; 18–25; 26–35; 36–45; 46–55; 56 and more; and rather not answer). Answers were recoded into three groups: 18–25, 26–35, and 36 and more. Underage participants were automatically redirected to the end of the questionnaire and excluded from the study. Two questions determined **gender identity**. Respondents indicated whether they identified as a man, woman, non-binary person, or other and whether their current gender identity differed from that assigned to them at birth. Answers were recoded into groups: women, men, and gender minorities. The term “gender minorities” refers to individuals who either did not identify as a man or woman or whose gender identity did not correspond to that assigned to them at birth; trans and non-binary individuals were thus included in “gender minorities.” Respondents indicated their **sexual identity** among eight possibilities, recoded into three groups: heterosexual, sexual minorities (homosexual, gay or lesbian; bisexual; two-spirited; queer, pansexual or allosexual; asexual), or uncertain/questioning. **Level of studies** was assessed using the following question: “Currently, what is your main status at the university?” We included only participants who answered “undergraduate student” and “graduate students” for the current study.” **International student status** was assessed using the following question: “Are you an international student (foreign student)?” Participants answered on a “yes/no” basis. **Disability status** was assessed using the following question: “Do you have a disability or health problem that impacts your daily life? This could be related to your physical condition, your mental health, or any other health condition.” Participants answered on a “yes/no/uncertain/rather not answer” basis. **Visible minority status** was assessed using the following question: “Do you consider yourself to be a “visible minority?” Visible minorities are people other than Indigenous people who do not identify themselves or are not perceived as white. Participants answered on a “yes/no/rather not answer” basis. **Indigenous status** was assessed using the following question: “Do you consider yourself part of an Indigenous community?” Participants answered on a “yes/no/rather not answer” basis on both questions on minority status. **Childhood sexual abuse** was assessed using two questions: Before the age of 18, “Has anyone ever touched you sexually when you did not want to or forced you to touch sexually anyone else?” and “Has anyone ever forced you to have sex (including oral, vaginal or anal penetration) when you did not want to?” Participants answered on a “yes/no/uncertain/rather not answer” basis.

#### Sexual Violence

In the context of the present study: “Sexual violence is defined as a sexual act that is committed or attempted by another person without freely given consent of the victim or against someone who is unable to consent or refuse.” (Basile et al., 2014, p. 11). This definition from the Center for Disease Control represents an umbrella term referring to multiple manifestations such as sexual assault, exhibitionism, voyeurism, sexual harassment, cyberstalking, unwanted touching, threats of rape, sexual blackmail, and various forms of unwanted or non-consensual sexual behavior. To measure sexual violence, a French translation of the Sexual Experiences Questionnaire (SEQ, Fitzgerald et al., 1999) was used. This 21-item scale was used to describe three dimensions of sexual violence: sexual harassment, unwanted sexual behaviors, sexual coercion, as well as total sexual violence. Sexual harassment was defined as verbal and nonverbal behaviors that include insults, hostile, and degrading attitudes without the goal of sexual cooperation. This variable was operationalized by eight questions (e.g., “repeatedly told you stories or sexual jokes that were offensive to you”; “made insulting or offensive comments with a sexual connotation”). Unwanted sexual behavior was defined as offensive, unwanted and non-reciprocal behaviors, including attempted rape and sexual assault. This variable was operationalized by seven questions (e.g., “touched you in a way that made you feel uncomfortable”; “had sex with you when you did not want to”). Sexual coercion was defined as a type of blackmail often involving promises of future considerations related to employment or studies. This variable was operationalized by five questions (e.g., “has caused you negative consequences because you have refused to engage in sexual activity”; “let you see that you would be rewarded in exchange for sexual favors”).

Respondents indicated on a five-point scale how many times each of the 21 items happened to them since arriving at university (never, once, two or three times, four or five times, and more than five times) and whether each occurred at least once over the last 12 months. Taking into account that the distribution was non-normal (i.e., the majority of respondents had not experienced sexual violence), a dichotomous score was computed for each dimension and a total score for their combined prevalence since arriving at university (0 = never, 1 = at least once since arriving at university). A past year incidence rate was computed for the three dimensions and their combination (0 = never, 1 = at least once over the last 12 months). Internal consistency was adequate for these three dimensions (0.84, 0.83, and 0.86, respectively) and the overall scale (0.89) (Bergeron et al., 2016).

#### Characteristics of University-Based Sexual Violence

Respondents who reported at least one event of UBSV answered a series of questions concerning the characteristics of the events. The first question concerned the contexts in which the events occurred (e.g., during an initiation, during class-related activities, online environment). The second set of questions documented the characteristics of the individuals who committed the UBSV (referred to as perpetrators): gender, status at the time of the event (e.g., student, professor, executive), and their hierarchical relationship to the respondent (inferior, equal, or superior). Multiple choice answers were possible for the context and the perpetrator’s characteristics, and all were recoded into dichotomous variables (yes/no).

### Data Analysis

Descriptive analyses were first conducted on all study variables to determine whether they distinguished varsity athletes from non-athletes (chi-square). The Sport Protection Hypothesis was tested using a series of logistic regressions on each dichotomous measure of violence. We included athlete status as a predictor and all sociodemographic characteristics associated with sexual violence victimization or distinguishing between varsity athletes and non-athletes as confounding variables. There were 0.3% of missing values for the overall data set. Less than 2% of participants had missing values for each variable, and 4% of respondents had at least one missing value. Such low missingness suggests imputation would bring negligible benefits (Schafer, 1999), specifically for logistic regressions, which are less susceptible to bias from missing data (Bartlett et al., 2015).

## Results

The chi-square analyses comparing the sociodemographic characteristics and the sexual violence against student-athletes and non-athletes are presented in Table 1. Varsity athletes were younger and more likely to be undergraduates, international and Indigenous students than their non-athlete peers. The effect sizes were moderate for age (Cramér’s *V* = 0.392) and international status (Cramér’s *V* = 0.369) and small for graduate level (Cramér’s *V* = 0.06) and Indigenous status (Cramér’s *V* = 0.133). All other characteristics (gender identity, sexual identity, disability status, visible minority status, childhood sexual abuse – CSA) had similar proportions among both groups. A higher proportion of varsity athletes reported sexual harassment. The proportion of varsity athletes and non-athletes reporting sexual violence since their arrival at university was similar for the three dimensions and their combination. Because there were no differences between athletes and non-athletes on the university prevalence of sexual violence since arriving at university, only the yearly incidence rates were used for the remaining analyses.

### Objective 1: Predicting Sexual Violence Against Varsity Athletes

Results of the four logistic regression analyses on the yearly incidence rates of the three dimensions of sexual violence and their combination using varsity athlete status as a predictor are presented in Table 2. Known predictors of sexual violence victimization on campus and participant characteristics that distinguish varsity athletes from non-athletes were included as confounding variables (age, graduate level, gender identity, sexual identity, disability status, visible minority status, international student status, Indigenous status, childhood sexual abuse). The four logistic regression models, including all predictors against a constant-only model, were statistically significant. For the combination of the three dimensions of sexual violence, the model explained 12.4% of the risk of exposure to sexual violence over the past year. The other three models explained 11.3% of the risk for sexual harassment, 8.7% of unwanted sexual behavior, and 6.9% of sexual coercion.

When considering all confounding variables in the four yearly incidence rates of sexual violence, the results revealed that being a varsity athlete did not significantly affect the risk of being exposed to sexual violence at university. All considered, other known risk factors were more significant predictors of the past year’s risk of sexual violence victimization than athlete status was. Overall, age and child sexual abuse were significant risk factors in all four models. Graduate level, gender identity, sexual identity, disability status, and international student status were risk factors in at least one model. Visible minority status and Indigenous status, like athlete status, did not reach significance levels in any models.

Many variables were associated with an increased risk for the past year’s combined forms of sexual victimization regardless of one’s varsity athlete status. Younger students, both for the 18–25 (3.06 times) and the 26–35 (2.02 times) categories were more at risk than those in the 36++++ category. Female students were 1.66 times more at risk than males. Participants who identified with the sexual minorities category were 1.83 times more at risk, while those who were uncertain or questioning of their sexual identity were at 1.90 times more at risk than heterosexual participants. International students were 1.45 times more at risk than domestic students. Students with a disability were 1.35 times more at risk than students who did not have disability. Finally, participants who were sexually abused as a child were 5.78 times more at risk of victimization and those who were uncertain or did not want to answer this question were 1.76 times more at risk than those who had not experienced CSA.

### Objective 2: Context of Violence Against Varsity Athletes Compared to Non-athletes

Analyses were conducted using only the sample of participants who reported at least one form of sexual violence against them (*n* = 2,307). Table 3 shows the results of the chi-squared analyses comparing the contextual characteristics of sexual violence of varsity athletes and non-athletes. Most students, including varsity athletes, reported that the perpetrator was another student. Whereas the reported proportion of other perpetrators was similar, varsity athletes were seven times more likely to have reported a sports coach than non-athletes (Cramér’s *V* = 0.130). About half of students, including varsity athletes, reported sexual violence during parties or social activities, excluding initiations or in a teaching context (Cramér’s *V* = 0.102). Varsity athletes were three times more likely to report sexual violence against them in the sporting context and ten times more likely during a sports initiation than non-athletes (Cramér’s *V* = 0.185). While these three contextual factors were significantly different between varsity athletes and non-athletes, the effect sizes are small.

**TABLE 3:** Sexual violence characteristics comparisons between varsity athletes and non-athletes reporting at least one form of sexual violence at university.

	Varsity Athletes	Non-athletes			
	*n* (%)	*n* (%)	X^2^	*p*	Cramér’s V
**Perpetrator status (*n* = 2,306)**					
Student	99 (90%)	1,866 (85%)	2.101	0.147	0.030
Teacher, director, supervisor	21 (19%)	423 (19%)	0.002	0.965	0.003
Sport coach	8 (7%)	18 (1%)	39.130	0.000	0.130
University staff excluding sport coach	9 (8%)	189 (9%)	0.24	0.877	0.003
Other (Client, patient, don’t know)	14 (13%)	319 (15%)	0.0274	0.600	0.011
**Perpetrator gender (*n* = 2,288)**					
Female	33 (30%)	621 (28%)	0.493	0.974	0.015
Male	101 (92%)	1,995 (91%)	0.757	0.944	0.018
Non-binary^a^	–	–			
**Hierarchical status of perpetrator (*n* = 2,290)**					
Inferior	6 (6%)	108 (5%)	0.80	0.777	0.006
Equal	96 (89%)	1,906 (87%)	0.221	0.638	0.010
Superior	29 (27%)	546 (25%)	0.183	0.669	0.009
**Context (*n* = 2,300)**					
Teaching context	49 (45%)	962 (44%)	0.46	0.830	0.004
During work, excluding teaching	12 (11%)	328 (15%)	1.293	0.255	0.024
During faculty or departmental initiation	23 (21%)	342 (16%)	2.345	0.126	0.032
Parties/social activities, excluding initiation	62 (57%)	1,183 (54%)	0.349	0.555	0.012
Sport context, excluding initiation	14 (13%)	77 (4%)	23.784	0.000	0.102
Sport initiation	11 (10%)	16 (1%)	78.437	0.000	0.185
Student involvement	18 (17%)	353 (16%)	0.012	0.911	0.002
Online	23 (21%)	416 (19%)	0.301	0.584	0.011
Other	17 (16%)	405 (19%)	0.578	0.447	0.016

*^a^Cell sizes for this category were too small for comparison.*

## Discussion

The purpose of the present study was to determine whether varsity athletes are at greater risk than non-athletes of being exposed to sexual violence or whether, on the contrary, involvement in varsity sport is coherent with the Sport Protection Hypothesis. Our results show that when controlling for potential confounding variables related to sexual violence victimization, being a varsity athlete did not significantly increase or reduce the risk of exposure to sexual violence at university overall and over the past year. All considered other variables were more significant predictors of the past year’s risk of sexual violence victimization than athlete status was. Namely, being younger, female, a sexual minority/questioning, an international student, having a disability, and having experienced child sexual abuse (CSA) were associated with an increased risk of the past year’s combined forms of sexual violence victimization, regardless of one’s varsity athlete status. These results align with prior research on these risk factors (Bergeron et al., 2016; Martin-Storey et al., 2018; Dion et al., 2021; Eisenberg et al., 2021; see text footnote 1; Klein and Martin, 2021). The proportion of the past year sexual violence rate that the models explain is less than 10%, indicating that other risk factors should be considered. Thus, our study’s results are not concordant with the Sport Protection Hypothesis nor with sport participation being particularly conducive to sexual violence victimization in the university context.

### Sport Protection Hypothesis

Our results are not concordant with the Sport Protection Hypothesis (Fasting et al., 2003) and previous work showing less sexual victimization in athletes (Fasting et al., 2008; Parent et al., 2016). In addition, the first explanation offered by Fasting et al. (2003) for the sport protection effect, namely that victims of sexual violence may be less likely to join a university sports program, is not supported by our results. We did not observe significant differences between varsity athletes and non-athletes regarding CSA victimization. The second explanation is related to the fact that sport in itself protects athletes from sexual victimization. Based on this second possibility, sport could be a protective factor against revictimization later in life, such as in the university context. However, our results do not support this explanation either. Indeed, while both athletes and non-athletes reported similar CSA rates, athletes were not less at risk of experiencing sexual violence in a university context than non-athletes.

### Sport as Risk Factor Hypothesis Based on Routine Activity Theory

If we cannot demonstrate support for the Sport Protection Hypothesis based on our results, we cannot support the hypothesis based on the Routine Activity Theory (Cohen and Felson, 1979). Following this theory, varsity athletes could be considered more at risk than other students on campus because they are in frequent contact with other male peer athletes, considered more probable perpetrators, and because of the institutional tolerance of sexual violence in sports (Jacobs et al., 2017; Parent and Fortier, 2018; Roberts et al., 2020). Despite previous evidence showing that being a member of a men’s university athletic team was a risk factor for sexual assault perpetration (Bonar et al., 2020), our results showed that varsity athletes were not more at risk of experiencing sexual violence when controlling for other important confounding variables. Based on this observation, we can postulate that interactions with potential perpetrators (i.e., male peer athletes) do not necessarily mean that other peer athletes become targets of those perpetrators. Previous findings were contradictory, with some showing that male athletes adhered to more rape myths and held more rape-supportive attitudes than non-athletes (Boeringer, 1999; Sawyer et al., 2002; Young et al., 2017), while not engaging in more sexual aggression behaviors than other groups in a university setting (Boeringer, 1996). Another study identified that membership in a sports team contributed to explaining only 1% of the variance in sexual assault in a university setting (Koss and Gaines, 1993). More research that would consider both the risk of victimization and of perpetration of varsity athletes could shed light on this issue.

### Explaining Risk of Sexual Violence in Athletes by Other Factors Than Varsity Athlete Status

When looking strictly at the incidence of sexual violence in the university context between varsity athletes and non-athletes, we observed that a higher proportion of varsity athletes reported sexual harassment (32 vs. 25%) and coercion (4 vs. 2%) over the past year, and more reported at least one form of sexual violence over the past year (35 vs. 27%). However, this increased yearly incidence rate seems to be primarily explained by other known risk factors. More specifically, as varsity athletes were younger, more likely to be undergraduates, Indigenous and international students, these differences may explain the higher uncorrected yearly rates. It does not appear to be because they were varsity athletes that they reported more sexual violence, but because they were more likely to report other known risk factors. This result means that sport participation is not a protective or a risk factor in our sample. The risk of experiencing sexual violence in a university context for varsity athletes might be interacting with individual and contextual factors unrelated to sport. Our results are in line with new research findings indicating that, in comparison to physical or psychological violence, sexual violence in sports is not very well explained by sport-specific factors, such as culture, norms, type of sport, level of sport, or hours of practice per week. Indeed, Vertommen et al. (submitted)^2^ recently showed that the main sport-factor predictors they used explained only 1% of sexual violence compared to 8% for physical violence, 10% for psychological violence and neglect, and 12% for instrumental violence. The results of Demers et al. (2021) also demonstrated that only 1.4% of sexual violence experienced by athletes (from peers) and 5.9% (from coaches) was explained by sport-specific social norms. In their work, (see text footnote 2) pointed out that more sexual violence-specific variables, such as social isolation or health problems, need to be included in future research looking at sexual violence in sports.

### Context and Perpetrator Profiles of Sexual Violence Experienced by Varsity Athletes

The second objective of our study was to determine whether the context and the profiles of perpetrators of sexual violence differed between varsity athletes and non-athletes. Our results showed that only three variables distinguished the two groups as varsity athletes were more likely than non-athletes to report: (1) sport coaches as perpetrators, (2) violence occurring in a sport context, and (3) a sport initiation context. Those results are not surprising as varsity athletes are more exposed to those contexts and perpetrators. However, our results show that only 7% of varsity athletes reported a coach as the perpetrator while most reported another student (90%). These proportions are similar to those of non-athletes in our study. Our data did not document whether student perpetrators were athletes and thus if varsity athletes were more likely than non-athletes to be victimized by another athlete. Previous studies have suggested this using varsity athlete samples where the most frequent perpetrators of sexual violence against them were peer athletes (Elendu and Umeakuka, 2011; Rintaugu et al., 2014). Further research could deepen our understanding of this question.

For both varsity athletes and non-athletes, most participants reported that the perpetrator’s gender was male, at 92 and 91% respectively. This is in line with previous research in sport (Bjørnseth and Szabo, 2018) and outside of sport (Dartnall and Jewkes, 2013; Tharp et al., 2013; Smith et al., 2017). A more surprising finding is that 30% of varsity athletes and 28% of non-athletes reported that the perpetrator gender was female. Research on sexual violence in sport involving female perpetrators is very limited. The study of Vertommen et al. (2017) pointed out that although male perpetrators were predominant, there was a notable proportion of female perpetrators in sport. Their results showed that 9% of athletes identified female perpetrators and 15% identified perpetrators of both sexes. This is supported by qualitative studies showing that female coaches or peer athletes could also be perpetrators (Johansson, 2018; Sanderson and Weathers, 2020). In another study, sport participation was identified as a risk factor for sexual harassment and sexual coercion in female high school students (Cheever and Eisenberg, 2022). The authors underlined that male students were proportionally more likely to be perpetrators but also pointed to the gap in the literature concerning athlete female-perpetrated sexual violence.

Similarly, results presented in our study show that Routine Activity Theory (RAT) was limited in explaining the risk of sexual violence for varsity athletes in university settings. Based on this theory we postulated that varsity athletes would be at increased risk of sexual victimization because they were potentially more in contact with male athletes and that male athletes’ status in university was associated with sexual violence beliefs and behaviors. However, we found that varsity athletes were victims of sexual violence not only by males but also by females, albeit in a lower proportion. This finding challenges the expectations set by RAT. The data collection did not include various contextual characteristics that would have been useful in furthering our understanding. For example, while participants identified the gender of the perpetrator, we did not ask their perpetrator’s athlete status. We also did not have details about the participants’ own athletic involvement, such as the type of sport practiced (team or individual, single or mixed sex). Those characteristics could help better understand the real risk posed by male varsity athletes towards other athletes for sexual violence in university settings. Finally, as Johansson (2018) stated, heteronormativity and predominant male perpetrator – female victim stereotypes may prevent looking closely at same-sex violence in sport. Results of some prevalence data in sport shows that female and male athletes are equally at risk of experiencing sexual violence (Parent et al., 2016; Parent and Vaillancourt-Morel, 2021), meaning that in sport, male to male sexual violence (or female to male) could be more frequent than expected. In sum, further studies should include more detailed contextual information that could allow comparing female and male perpetrators in terms of types of sexual violence committed and the related contextual characteristics.

In terms of context, our results show that varsity athletes were three times more likely to report sexual violence occurring in the sporting context and ten times more likely during a sports initiation than non-athletes. However, about half of students, including varsity athletes, reported being sexually victimized during parties or social activities. It is possible that varsity athletes also experienced sexual violence in sport-related parties or social activities. However, our study did not document this level of contextual detail as it was not specifically designed for athletes.

### Strengths, Limitations, and Future Research

The current study has several strengths. Most importantly, it is the first to our knowledge that compares varsity athletes with non-athletes’ risk of sexual violence on campus. Moreover, these comparisons were conducted while controlling known risk factors for sexual violence in a university context to account for potential confounding variables. We were also able to document in a more clearly delineated manner sexual violence before becoming a varsity athlete (childhood sexual abuse) and sexual violence while at university (since entering university and last year). This improves on previous tests of the Sport Protection Hypothesis. However, some limitations should also be noted. Although the convenience sample was large and diverse, it did not represent all university students. Moreover, it is possible that students who had previously been abused had abandoned their participation in sports. This would contribute to decreasing the overall prevalence rates for varsity athletes as leaving sport is one of the consequences of sexual violence reported by athletes (Fasting et al., 2002; Fogel and Quinlan, 2021). Another limitation of our study is that our questionnaire did not provide information on the type of sport practiced by varsity athletes, which prevents us from carrying out analyses according to the types of sports practiced. Finally, the study prevented the inclusion of students who might be athletes outside of university context. It would have been pertinent to include in our analyses these athletes who are not part of varsity teams but might still have experienced sexual violence at their university.

In conclusion, while the current study is helpful to deepen our understanding of varsity athletes’ risk of sexual violence in the university context, much remains to be explored. The analyses revealed that the context of varsity sport might not confer protection against or represent a risk factor for all athletes. As such, the study’s findings have cast doubt on the Sport Protection Hypothesis and that the varsity sport environment is conducive to sexual violence, which we hypothesized based on prior research results and the Routine Activity Theory (Cohen and Felson, 1979). Further investigations should be conducted to determine if subgroups of varsity athletes are more at risk of sexual violence victimization. Our findings indicate we need more information on varsity athlete subgroups such as undergraduates, Indigenous, and international students to determine if they are more at risk and, if so, why. Further analyses of potential interactions among these intersecting risk factors and varsity athlete status need to be conducted. This could help develop adequate and specific preventative measures. A longitudinal study that would track participants from their teenage years onward would also allow for more robust evidence of the impact of sport participation. Such study would indicate whether sport participation represents a protective factor against sexual violence later in life or a context conducive to developing rape-supportive attitudes and behaviors.

## Data Availability Statement

The data analyzed in this study is subject to the following licenses/restrictions: The dataset can be made available upon request. Requests to access these datasets should be directed to MB, bergeron.manon@uqam.ca.

## Ethics Statement

The studies involving human participants were reviewed and approved by Comité institutionnel d’éthique de la recherche avec des êtres humains de l’Université du Québec à Montréal Comité d’éthique de la recherche en arts et en sciences de l’Université de Montréal Comité sectoriel d’éthique de la recherche en psychologie et en sciences de l’éducation de l’Université Laval Comité d’éthique de la recherche de l’Université du Québec à Chicoutimi Comité d’éthique de la recherche de l’Université du Québec en Outaouais Comité d’éthique de la recherche Éducation et sciences sociales de l’Université de Sherbrooke. The patients/participants provided their written informed consent to participate in this study.

## Author Contributions

SP contributed to the conception and study design and drafted the manuscript. ID contributed to the conception and study design, conducted the analyses, and reviewed the manuscript. SR contributed to the analyses and reviewed the manuscript. MB was the lead investigator on the study from which the data originated and contributed to the conception, study design, and project management of the initial study, and reviewed the current manuscript. All authors made substantial contributions to the manuscript, approved the final version before its publication, and agreed to be accountable for the content of the work.

## Conflict of Interest

The authors declare that the research was conducted in the absence of any commercial or financial relationships that could be construed as a potential conflict of interest.

## Publisher’s Note

All claims expressed in this article are solely those of the authors and do not necessarily represent those of their affiliated organizations, or those of the publisher, the editors and the reviewers. Any product that may be evaluated in this article, or claim that may be made by its manufacturer, is not guaranteed or endorsed by the publisher.

## Footnotes

1 Fethi, I., Daigneault, I., Bergeron, M., Hébert, M., and Lavoie, F. (under review). Campus sexual violence victimization: a comparison of international and domestic students. *J. Int. Stud.*

2 Vertommen, T., Decuyper, M., Parent, S., Pankowiak, A., and Woessner, M. N. (submitted). Interpersonal violence in Belgian sport today: young athletes report. *J. Sport Soc. Issues*
